# Development of a primary care screening algorithm for the early detection of patients at risk of primary antibody deficiency

**DOI:** 10.1186/s13223-023-00790-7

**Published:** 2023-05-27

**Authors:** Marianne A. Messelink, Roos M. Berbers, Joris M. van Montfrans, Pauline M. Ellerbroek, André Gladiator, Paco M. J. Welsing, Helen Leavis

**Affiliations:** 1 Department of Rheumatology & Clinical Immunology, University Medical Center Utrecht, Heidelberglaan 100, 3508 GA Utrecht, The Netherlands; 2 Takeda Pharmaceuticals International AG, Thurgauerstrasse 130, 8152 Glattpark-Opfikon, Zurich, Switzerland

**Keywords:** Diagnostic delay, Primary antibody deficiency, Algorithm, Screening, Primary care, Electronic health record

## Abstract

**Background:**

Primary antibody deficiencies (PAD) are characterized by a heterogeneous clinical presentation and low prevalence, contributing to a median diagnostic delay of 3–10 years. This increases the risk of morbidity and mortality from undiagnosed PAD, which may be prevented with adequate therapy. To reduce the diagnostic delay of PAD, we developed a screening algorithm using primary care electronic health record (EHR) data to identify patients at risk of PAD. This screening algorithm can be used as an aid to notify general practitioners when further laboratory evaluation of immunoglobulins should be considered, thereby facilitating a timely diagnosis of PAD.

**Methods:**

Candidate components for the algorithm were based on a broad range of presenting signs and symptoms of PAD that are available in primary care EHRs. The decision on inclusion and weight of the components in the algorithm was based on the prevalence of these components among PAD patients and control groups, as well as clinical rationale.

**Results:**

We analyzed the primary care EHRs of 30 PAD patients, 26 primary care immunodeficiency patients and 58,223 control patients. The median diagnostic delay of PAD patients was 9.5 years. Several candidate components showed a clear difference in prevalence between PAD patients and controls, most notably the mean number of antibiotic prescriptions in the 4 years prior to diagnosis (5.14 vs. 0.48). The final algorithm included antibiotic prescriptions, diagnostic codes for respiratory tract and other infections, gastro-intestinal complaints, auto-immune symptoms, malignancies and lymphoproliferative symptoms, as well as laboratory values and visits to the general practitioner.

**Conclusions:**

In this study, we developed a screening algorithm based on a broad range of presenting signs and symptoms of PAD, which is suitable to implement in primary care. It has the potential to considerably reduce diagnostic delay in PAD, and will be validated in a prospective study.

*Trial registration* The consecutive prospective study is registered at clinicaltrials.gov under NCT05310604

**Supplementary Information:**

The online version contains supplementary material available at 10.1186/s13223-023-00790-7.

## Background

Primary antibody deficiencies (PAD) are characterized by an inability to produce clinically effective immunoglobulin responses and represent the majority of all primary immunodeficiency (PID) disorders [1–3]. PAD encompass a heterogeneous group of diseases such as common variable immunodeficiency (CVID), X-linked agammaglobulinemia (XLA), immunoglobulin (Ig) G subclass deficiency and specific antibody deficiency (SpAD) [4]. The estimated prevalence of PAD varies widely from 1:700 to 1:25.000, in part due to the suspected large number of undiagnosed patients [5–8].

The onset of symptoms of PAD is most commonly in the second to fourth decade of life [9–12]. The clinical presentation is heterogeneous, including increased susceptibility for respiratory tract and gastro-intestinal infections, auto-immune symptoms, lymphoproliferative disease and an increased risk of certain malignancies [4, 13–15]. Owing to the wide variety in presenting symptoms and the rarity of PAD, diagnosis can be challenging. This is evident by the reported mean diagnostic delay of 6–12 years (median 3–10 years), which has not significantly improved over the past 5 decades [13, 16–18]. This diagnostic delay may increase the risk of morbidity and mortality, as effective therapies and prophylaxes are available [18–21]. A timely diagnosis may also lead to substantial health care cost savings, even when correcting for the cost of treatment [22]. Thus, reducing the diagnostic delay of PAD is of key interest [18].

To this end, several ‘Early warning signs’ sets have previously been developed. However, these do not include the full spectrum of presenting symptoms of PAD, show suboptimal diagnostic performance, and are often used in a non-automated manner [23–28]. As manual screening systems depend on the awareness of an individual physician, these are suboptimal for rare diseases with heterogeneous presentations. Therefore, we aimed to develop a screening algorithm that encompasses an extended spectrum of PAD signs and symptoms based on electronic health record (EHR) data, that can easily be automated. Such an algorithm may be used as an aid to notify general practitioners (GPs) when further laboratory evaluation of immunoglobulins should be considered, thereby facilitating a timely diagnosis of PAD.

Primary care may be the optimal setting for such an algorithm for several reasons. Firstly, a patient will initially present with their symptoms in primary care, especially in countries where the GP has a gatekeeper function to secondary care. Therefore, screening in primary care could allow detection of PAD patients in an earlier phase when compared to screening in secondary care. Secondly, primary care EHRs encompass a comprehensive overview of the complaints for which a patient has sought medical care. In contrast, in secondary care usually only the symptoms for which a patient has been referred are registered structurally (i.e. in diagnostic codes). For example, if a patient is only referred for respiratory tract infections, relevant gastro-intestinal or auto-immune symptoms could be missed. Thus, focusing on primary care allows screening for a broad range of presenting signs and symptoms of PAD.

The aim of the current study is to develop a screening algorithm to identify patients with an increased risk of PAD in a primary care setting. This algorithm could be applied to notify GPs of patients at risk of PAD, for whom further laboratory investigation of immunoglobulins and/or consultation of an immunologist is indicated.

## Methods

The algorithm for the early detection of PAD was developed based on primary care EHR data of PAD patients and control groups. We focused on data that are available as structured data in the EHRs of primary care facilities in the Netherlands. Almost all inhabitants of the Netherlands have an EHR with a public primary care physician, and the primary care physician has a gatekeeper function to secondary care, signifying that in non-urgent cases referral from the GP is necessary to access hospital care [29]. Available structured data included age, diagnostic ICPC codes (International Classification of Primary Care), ATC codes (Anatomical Therapeutic Chemical) for medication prescriptions and (requests for) laboratory assessments.

**Fig. 1:**
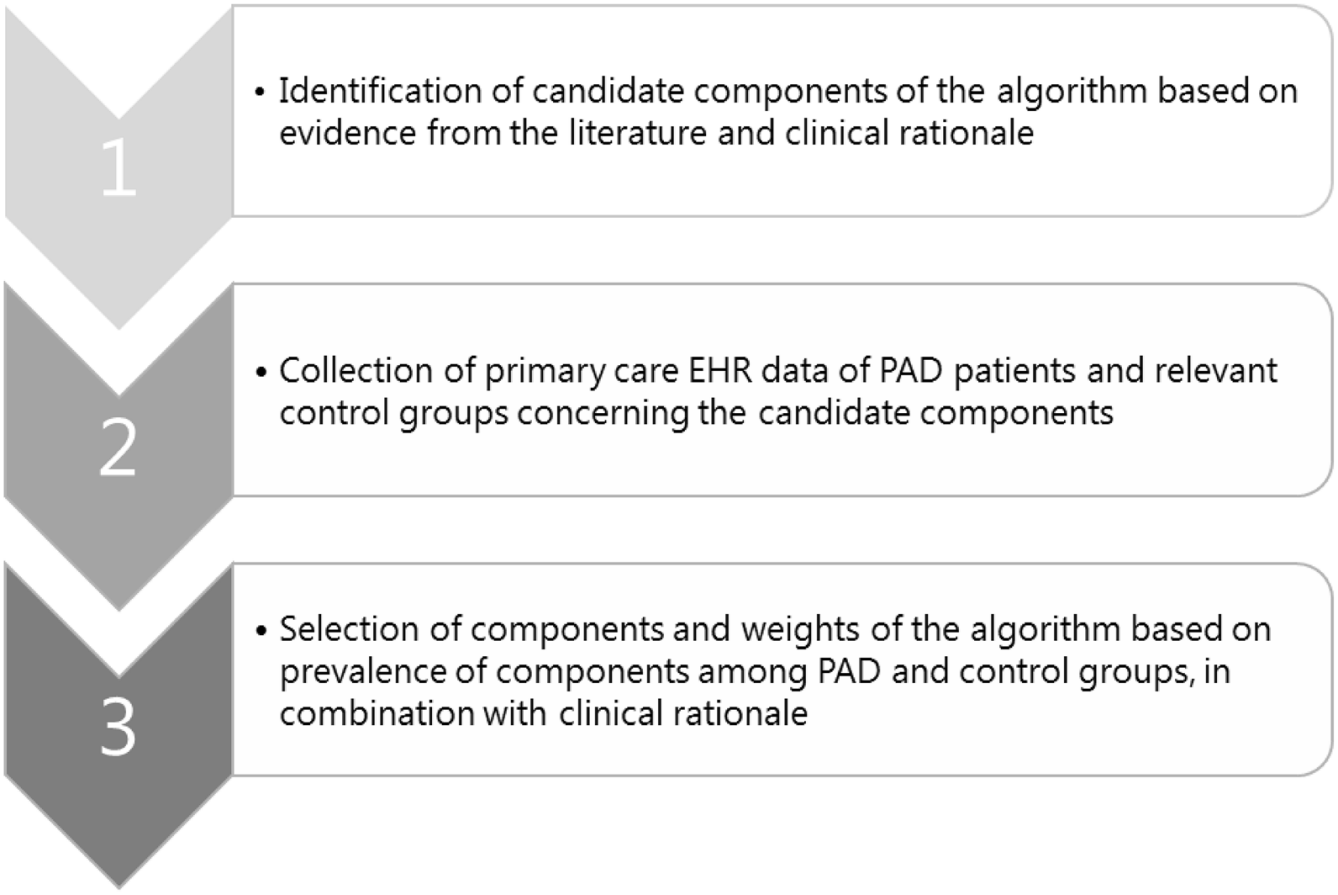
Steps in the development of the screening algorithm for primary antibody deficiencies. *EHR* electronic health record, *PAD* primary antibody deficiencies

The algorithm was developed in 3 steps, see Fig. 1.

### Identification of candidate components of the algorithm

The hallmark clinical features of PAD are recurrent respiratory tract infections (RTI), including otitis media, sinusitis and pneumonia [12, 21, 30]. Gastro-intestinal complaints, such as chronic diarrhea and parasitic infections, also occur frequently in certain PAD [12, 31–34]. In addition, it is estimated that around 30% of PAD patients develop auto-immune disorders, e.g. idiopathic thrombocytopenic purpura, auto-immune hemolytic anemia or rheumatoid arthritis [35–37]. Finally, certain PAD can also present with lymphoproliferative symptoms and are associated with an increased risk of non-Hodgkin lymphoma and gastric cancer [38–41]. All diagnostic ICPC codes related to these presenting symptoms of PAD were considered as candidate components of the algorithm.

Since PAD patients often present with recurrent infections that require antibiotic treatment, we also considered the type and number of antibiotic prescriptions for inclusion in the algorithm. We considered antibiotics registered in the Netherlands for the treatment of respiratory tract or parasitic intestinal infections [42]. As antibiotics for upper RTIs are frequently prescribed in the early years of childhood, we only took prescriptions from the age of 6 years into account [43].

As PAD, by definition, can be characterized by a reduced level of one or more (subtypes of) immunoglobulins, we considered these laboratory results for inclusion in the algorithm. Since immunoglobulin diagnostics may not be requested commonly in primary care, we also considered calculated globulin. Calculated globulin is a measure based on total protein and albumin levels, which can indicate hypogammaglobulinemia [44, 45].

A previous study reported that a higher number of consultations at the GP, as well as a higher number of requests for lung function tests and blood tests for infection, were statistically significantly associated with an increased odds of CVID diagnosis [46]. Therefore, we also considered these factors for inclusion in the algorithm.

In contrast to PAD, secondary antibody deficiencies are the consequence of an underlying disease, such as hematological malignancy. Diagnoses known to be causes of secondary antibody deficiencies (e.g. leukemia, multiple myeloma, HIV) were added as exclusion criteria [47–49]. In the case that a diagnosis could be both a cause of secondary antibody deficiency as well a complication of PAD (e.g. non-Hodgkin lymphoma), it was labelled as ‘‘ambiguous diagnosis’’. For these diagnoses, the EHRs were screened up to the moment of the ambiguous diagnosis. This approach allows detection of patients for whom the ambiguous diagnosis is the consequence of an underlying PAD, whilst still excluding patients with a secondary antibody deficiency. Immunosuppressant medication was not added as an exclusion criterion, as these may also be prescribed for auto-immune symptoms that are caused by an underlying PAD. Table 1 shows an overview of the candidate components of the algorithm.

**Table 1:** Overview of candidate components from primary care electronic health records

Type of electronic health record data	Description
ICPC codes	Diagnostic codes related to presenting signs and symptoms of PAD For exclusion criteria: diagnostic codes related to causes of secondary antibody deficiencies
ATC codes	Antibiotic prescriptions for the treatment of respiratory tract or parasitic infections
Laboratory results	IgA, IgM, IgG (total and subclasses), calculated globulin
Number of visits	Number of physical or telephone appointments with the general practitioner
Number of requests for additional tests	Number of requests for CRP, leukocytes and lung function tests

Overview of candidate components for the algorithm as extracted from structured primary care EHR data

*ATC* Anatomical Therapeutic Chemical, *CRP* C-reactive protein, *EHR* electronic health record, *ICPC* International Classification of Primary Care, *Ig* immunoglobulin, *PAD* primary antibody deficiencies

### Collection of EHR data of PAD patients and control groups

To gain insight in the prevalence of the candidate algorithm components among PAD patients and control groups, we used two data sources. First, we collected the primary care EHR data of 30 patients from different age groups with a variety of PAD diagnoses from an academic hospital in the Netherlands (the University Medical Center Utrecht). Patients signed informed consent and research agreements were signed by their GPs. Second, we collected primary care EHR data from the pseudonymized database of the Julius General Practitioner Network (JHN), a research collaboration of GPs in the region of Utrecht, Netherlands [50]. Owing to privacy regulations from the JHN database, we only had access to the metadata (e.g. means, medians and proportions) concerning the candidate components of the algorithm for several patient groups of interest, defined based on registered diagnostic ICPC codes. The groups of interest included PAD patients as well as several control groups. Because no specific ICPC code for PAD exists, we selected the first group based on the code for immunodeficiency (T99.01). This group approximates PAD patients, as causes of secondary immunodeficiencies were excluded based on the exclusion criteria, and because PAD represents the majority of the remaining PIDs. Therefore, this group of ‘primary care immunodeficiency’ patients was considered to be of interest, in addition to the group of confirmed PAD-patients from the academic hospital.

The selected control groups were ‘patients from the general GP population’, ‘patients with upper RTIs’, ‘patients with chronic obstructive pulmonary disease (COPD) or asthma’, ‘patients with inflammatory bowel disease (IBD)’ and ‘patients with a malignancy’. These groups were selected as they were expected to have overlapping presenting symptoms with PAD patients, but should be distinguished from potential PAD patients by the algorithm in order to prevent false-positives. For all control groups we applied the exclusion criteria, and excluded patients that had an ICPC code for immunodeficiency (T99.01). The meta-data of all available patients in the JHN-database that met the criteria for the control groups were utilized.

From both the academic hospital and the primary care database we selected patients aged 12–70 years, as PAD most commonly presents in the second to fourth decade of life [9–12]. In addition, as recurrent infections and antibiotic prescriptions are common in younger children, other cut-off values are to be expected for this age group. Lastly, our algorithm was developed in line with the study design of a subsequent external validation study, where high-risk patients will be invited for laboratory analysis of immunoglobulins. Considering the medical ethical guidelines, this validation study is only feasible in patients aged ≥ 12 years.

A distinct ‘censoring date’ was selected for different patient groups. All available EHR data before the censoring date were extracted. The censoring date indicates the date up to which time the EHR data were extracted. For most patients, this was the date of data-extraction, November 18, 2021. For the PAD and primary care immunodeficiency patients, we extracted the EHR data both pre- and post-diagnosis. The censoring date could therefore be the date of PAD diagnosis (pre-diagnosis), or the date of data-extraction (post-diagnosis). When deciding which components were to be maintained in the algorithm, pre-diagnosis data were used because our aim is to identify patients before diagnosis. For patients with an ambiguous diagnosis, the date of this specific diagnosis was defined as their censoring date.

To determine the validity of the diagnostic ICPC codes, we compared the presenting symptoms registered as diagnostic ICPC codes in the primary care EHR of the PAD patients with the presenting symptoms registered as free text in the secondary care EHR of the academic hospital and determined their concordance.

### Selection of components and weights of the algorithm

The decision to include a candidate component in the algorithm and the corresponding weight was based on the results of analyses of the corresponding EHR data in combination with clinical rationale. To estimate the ability of the presence of individual diagnostic ICPC codes to differentiate PAD patients from the control group, we calculated the Youden’s index. Youden’s index is a summary measure of diagnostic quality based on sensitivity and specificity, where a higher index indicates that the component has a better discriminatory ability [51]. The sensitivity of the presence of a component was calculated for the PAD patients and primary care immunodeficiency patients, and the mean of the sensitivities the was used to determine Youden’s index. Specificity was calculated from the general GP population control group. The weight of the diagnostic ICPC codes was based on the discriminative value as expressed by Youden’s index in combination with clinical rationale.

The diagnostic ICPC codes included in the algorithm were grouped according to the following categories: ‘respiratory tract infections’, ‘gastro-intestinal complaints’, ‘other infections’, ‘auto-immune symptoms’ and ‘malignancies, lymphoproliferative and other symptoms’. For each of these groups, we determined whether the entire EHR before the censoring date should be screened for these diagnostic codes, or only the 10 years before the censoring date. This decision was based on clinical rationale and on the discriminative value as expressed by Youden’s index.

For the ATC codes, we calculated the mean number of antibiotic prescriptions per year in the 10 years before the censoring date. The decision on the inclusion and weight of individual antibiotics was based on both clinical rationale and the difference in the mean number of prescriptions between the control groups and immunodeficiency patients.

For immunoglobulin levels and calculated globulin, we determined the mean laboratory values in the EHR in the 10 years before the censoring date. Lastly, regarding the number of consultations at the GP and the number of requests for lung function tests/blood tests for infection, we calculated the mean and median number of visits and requests in the year before the censoring date for both the PAD/primary care immunodeficiency groups and the control groups. To determine the optimal cut-off point for the number of lung function tests, blood tests for infection and number of visits to the GP, we calculated the Youden’s index for several cut-off points spread around the median values in PAD patients.

Ethical approval for this study was received from the Medical Research Ethics Committee Utrecht, protocol number 19–748. Statistical analysis was done with the base package in R version 4.0.2.

## Results

### Patient characteristics

Of the 30 included PAD patients from the academic hospital, 12 were diagnosed with CVID, 8 with IgG subclass deficiency, 4 with unclassified antibody deficiency, 3 with IgA and IgG subclass deficiency, 2 with selective IgA deficiency and 1 patient was diagnosed with SpAD. The mean age at the time of data extraction was 29.8 years, with a large variation (standard deviation of 14). The PAD patients thus represented a wide range of diagnoses and ages. The mean delay from symptom onset to diagnosis of these patients was 12.4 years [SD 12.2, median 9.5 (3.0–19.5)]. The patient characteristics of the PAD patients from the academic hospital, of the primary care immunodeficiency patients and the control groups (all aged 12–70 years) are shown in Table 2.

**Table 2:** Patient characteristics

Group	No. of patients	% Female	Mean age (SD)
General population	58,223	52.9	39.8 (13.9)
Upper RTI	13,133	57.4	38.4 (16.3)
COPD/asthma	4427	53.2	42.3 (15.7)
IBD	403	54.6	43.8 (12.9)
Malignancy	1526	60.5	55.2 (12.0)
Primary care immunodeficiency	26	53.8	At diagnosis: 33.3 (15.4)
			At data-extraction date: 40 (14.8)
PAD	30	40	Onset symptoms: 6.4 (7.4)
			At diagnosis: 19.4 (16.7)
			At data-extraction date: 29.8 (14)

Patient characteristics of the different control groups, the immunodeficiency patients registered in primary care and the PAD patients registered in an academic hospital. If not stated otherwise, the values were captured on the date of data extraction (November 2021)

*COPD* chronic obstructive pulmonary disease, *IBD* inflammatory bowel disease, *JHN* Julius General Practitioner Network, a general practitioner network in Utrecht, Netherlands, *no.* number, *RTI* respiratory tract infection, *SD* standard deviation

### Diagnostic ICPC codes

Table 3 shows the concordance between the presenting symptoms registered as diagnostic ICPC codes in the primary care EHRs and the symptoms registered in free text in the secondary care EHRs. Concordance was assessed by calculating the percentage of patients for whom the presence or non-presence of a symptom was equal between the diagnostic codes in the primary care EHR and the free text in the secondary care EHR. For almost all presenting symptoms, apart from gastro-intestinal complaints, concordance between the diagnostic primary care ICPC codes and the free text in secondary care EHRs was high.

**Table 3:** Concordance between PAD symptoms registered as diagnostic ICPC codes and free text

Presenting symptom	Registered in primary care EHR (diagnostic codes)	Registered in secondary care EHR (free text)	Concordance (%)
Upper RTI	30 (100%)	28 (93%)	93
Gastro-intestinal symptoms	17 (57%)	12 (40%)	57
Pneumonia	11 (37%)	11 (37%)	87
Auto-immune symptoms	11 (37%)	9 (30%)	87
Bronchiectasis	6 (20%)	6 (20%)	87
Arthritis/arthralgia	0 (0%)	4 (13%)	87
Meningitis	4 (13%)	4 (13%)	100

Concordance between symptoms registered as diagnostic codes in the primary care EHR and symptoms registered as free text in the secondary EHR. Data of 30 patients of an academic hospital before their PAD diagnosis. Data in columns 2 and 3 are presented as number of patients (%)

*EHR* electronic health record, *ICPC* International Classification of Primary Care, *PAD* primary antibody deficiency, *RTI* respiratory tract infection

The selection of diagnostic codes and their attributed weight was based on the Youden’s index (Additional file 1: Table S1) in combination with evidence from literature and clinical expertise of PAD specialists in the University Medical Center Utrecht. The diagnostic codes included in the final algorithm are shown in Table 5.

### Antibiotic prescriptions

**Fig. 2:**
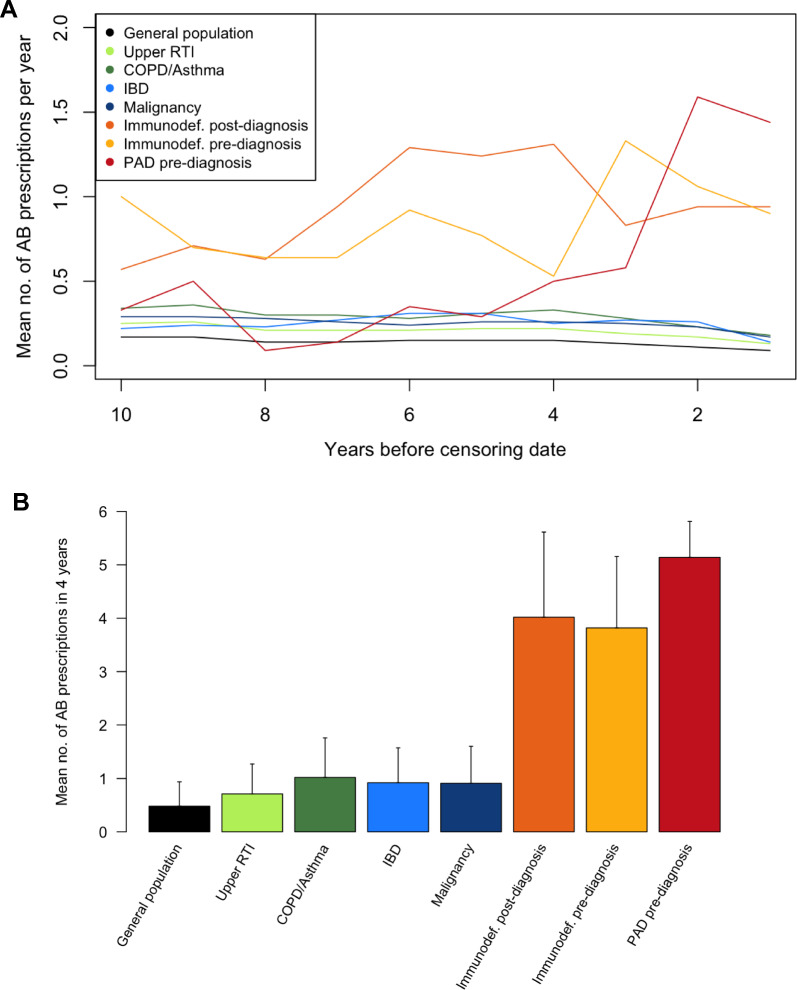
**A** Mean number of antibiotic prescriptions per year in the 10 years before the censoring date. **B** Mean total number of antibiotic prescriptions in 4 years before the censoring date. *AB* antibiotic, *COPD* chronic obstructive pulmonary disease, *IBD* inflammatory bowel disease, *immunodef.* immunodeficiency patients from primary care, *no.* number, *PAD* primary antibody deficiencies patients from an academic hospital, *RTI* respiratory tract infection

Figure 2A shows the mean number of antibiotic prescriptions per year in the 10 years before the censoring date for the different patient groups. PAD patients and primary care immunodeficiency patients had a higher mean number of antibiotic prescriptions than all control groups. In the pre-diagnosis groups this effect appeared to be most pronounced from 4 years before the diagnosis. Figure 2B shows the mean total number of antibiotic prescriptions in the 4 years before the censoring date, where the immunodeficiency and PAD groups had a higher mean compared to the control groups (5.14 in PAD vs. 0.48 in the general population). It was therefore decided to include antibiotic prescriptions from the past 4 years in the algorithm, see Table 5. The mean number of antibiotic prescriptions per individual antibiotic is shown in Additional file 2: Table S2. The weight of the individual antibiotics was based on the difference in means between immunodeficiency and control groups in combination with clinical rationale. For example, if the difference in means was < 0.05, we generally attributed a weight of 1, rather than a weight of 2, for each antibiotic prescription.

### Laboratory results and number of requests

Table 4 shows the mean laboratory values of the immunoglobulins and calculated globulin in the EHR in the 10 years before the censoring date. Immunoglobulins were only requested for a very small proportion of patients from the control groups. Calculated globulin was requested more often than immunoglobulins in these control groups, although still for a very small proportion. For example, calculated globulin was requested only for 0.38% of the general GP population. For the PAD and primary care immunodeficiency patients, immunoglobulins and calculated globulin were not requested pre-diagnosis. Although these parameters are thus not likely to apply to many patients, a reduced level of immunoglobulins or calculated globulin was still added to the algorithm based on the strong clinical relevance. Although it could be expected that a PAD diagnosis is quickly made after immunoglobulin testing, patients may be missed by the GP, thus making this a relevant component to add to the screening algorithm.

**Table 4:** Mean laboratory values of immunoglobulins in the 10 years before the censoring date

	General population (N = 58,223)	Upper RTI (N = 13,133)	COPD/asthma (N = 4427)	IBD (N = 403)	Malignancy (N = 1526)	Immunodef. pre-diagnosis (N = 26)	PAD pre-diagnosis (N = 30)
IgA (g/L)	2.01 (1.06) (n = 541)	2.09 (1.11) (n = 240)	2.32 (1.44) (n = 63)	2.51 (1.00) (n = 10)	2.31 (0.98) (n = 24)	–	–
IgG1 (g/L)	3.8 (0) (n = 1)	3.8 (0) (n = 1)	–	–	–	–	–
IgG2 (g/L)	3.34 (0) (n = 1)	3.34 (0) (n = 1)	–	–	–	–	–
IgG3 (g/L)	0.67 (0) (n = 1)	0.67 (0) (n = 1)	–	–	–	–	–
IgG4 (g/L)	0.06 (0) (n = 1)	0.06 (0) (n = 1)	–	–	–	–	–
IgG total (g/L)	10.6 (2.87) (n = 65)	11.28 (2.09) (n = 34)	11.08 (1.55) (n = 11)	–	11.37 (1.96) (n = 9)	–	–
IgM (g/L)	2.63 (4.73) (n = 67)	1.79 (1.97) (n = 34)	2.44 (9.53) (n = 11)	–	2.49 (6.42) (n = 10)	–	–
Calculated globulin (g/L)	26.34 (12.12) (n = 221)	30.66 (9.25) (n = 89)	26.79 (13.53) (n = 38)	33.70 (0.71) (n = 3)	28.61 (15.83) (n = 23)	–	–

Values are shown as mean (standard deviation). EHR data from the 10 years before the censoring date. A ‘–’ indicates that this laboratory value has not been requested for this patient group

*COPD* chronic obstructive pulmonary disease, *IBD* inflammatory bowel disease, *EHR* electronic health record, *Ig* immunoglobulin, *immunodef.* immunodeficiency patients from primary care, *PAD* primary antibody deficiencies, *RTI* respiratory tract infection

Based on previous research by Ilkjær et al. [46] we also considered the number of requests of leukocytes, C-reactive protein and lung function tests by the GP. These however did not appear to have a discriminatory value when comparing the general population with immunodeficiency patients, see Additional file 3: Fig. S1. These components were thus not added to the algorithm.

### Visits to the general practitioner

**Fig. 3:**
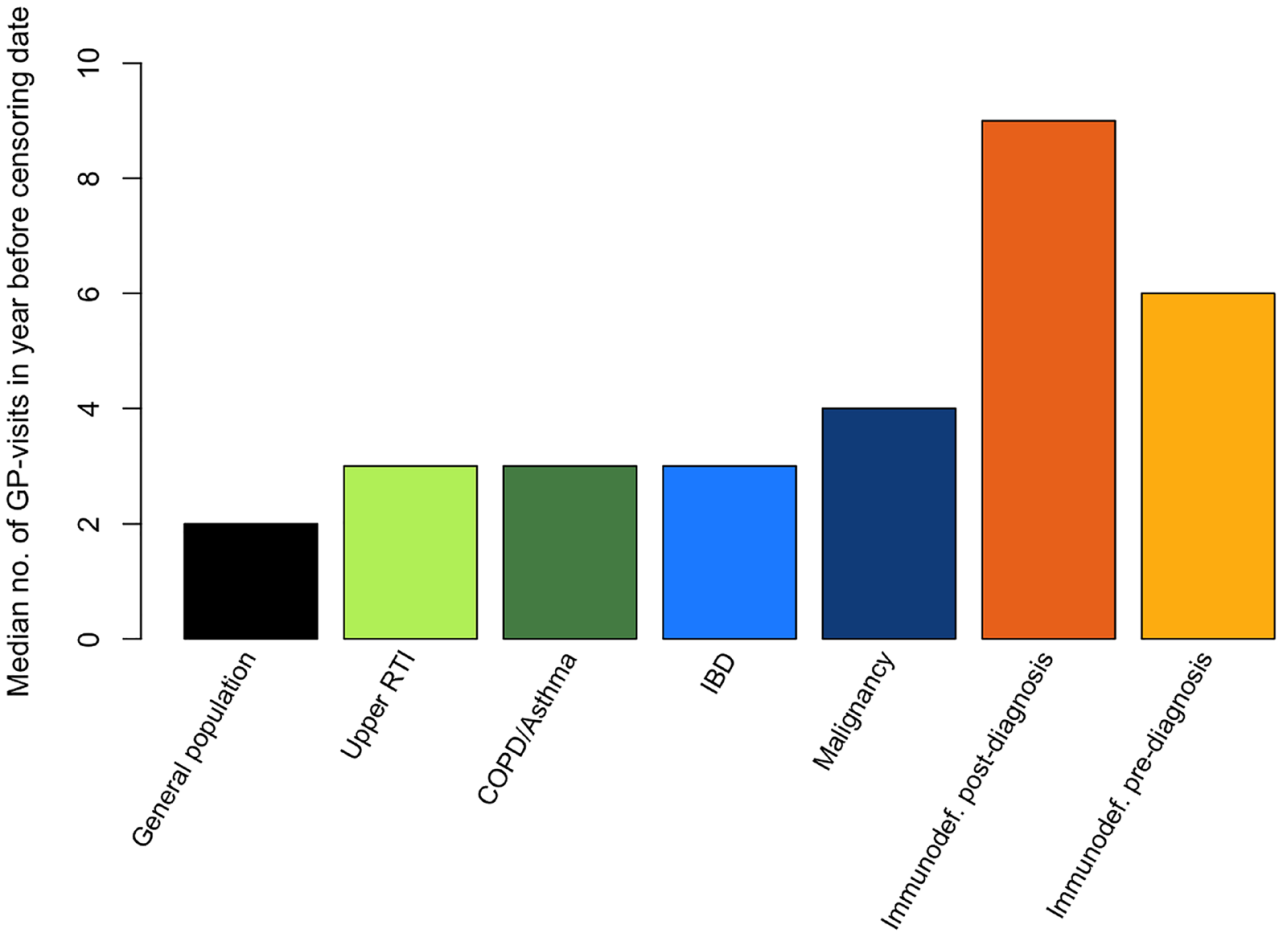
Median number of visits to the general practitioner in the year before the censoring date. Both physical and electronic visits are included. *COPD* chronic obstructive pulmonary disease, *GP* general practitioner, *IBD* inflammatory bowel disease, *immunodef.* immunodeficiency patients from primary care, *no.* number, *RTI* respiratory tract infection

Figure 3 shows the median number of visits to the GP in the year before the censoring date. The immunodeficiency groups showed a higher median number of visits to the GP compared to the control groups. For example, PAD patients pre-diagnosis had a median of 6 visits per year, compared to 2 visits per year in the general population. These data were not available for the PAD patients from the academic hospital. Based on Youden’s index the cut-off value for the algorithm was set to ≥ 6 visits to the GP in a year.

### Algorithm

The components and corresponding weights of the final PAD screening algorithm are shown in Table 5. The categories of the algorithm include ‘Antibiotics’, ‘Respiratory tract infections’, ‘Gastro-intestinal complaints’, ‘Other infections’, ‘Auto-immune symptoms’, ‘Malignancies, lymphoproliferative and other symptoms’, ‘Laboratory values’ and ‘Visits to general practitioner’.

**Table 5:** Screening algorithm for early detection of primary antibody deficiencies in a primary care setting

Antibiotics^a^		
ATC-code	Description	Score per prescription
J01AA02	Doxycycline	2
J01CA04	Amoxicillin	2
J01CF05	Flucloxacillin	1
J01CR02	Amoxicilline/clavulanic acid	2
J01EE01	Cotrimoxazole	2
J01FA01	Erythromycin	2
J01FA09	Clarithromycin	2
J01FA10	Azithromycin	2
J01MA02	Ciprofloxacin	1
J01MA12	Levofloxacin	2
J01MA14	Moxifloxacin	2
P01AB01	Metronidazole	1
J01CE05	Pheneticillin	2
J01CE02	Phenoxymethylpenicillin	2
J01DB01	Cefalexin	2
J01DC04	Cefaclor	2
J01DD14	Ceftibuten	2
J01DC02	Cefuroximaxetil	2
S02CA03	Hydrocortisone/colistin/bacitracin ear suspension	0.5
S02AA16	Ofloxacin ear suspension	0.5

ICPC code	Description	Score for presence of code
Respiratory tract infections^b^		
H01	Ear pain	1
H04	Discharge from ear	1
H71	Acute otitis media/myringitis	2
H72	Otitis media with effusion	2
H74	Chronic otitis media/other ear infections	1
H74.01	Chronic otitis media	1
H74.02	Mastoiditis	2
R05	Coughing	1
R07	Sneezing/nasal congestion/running nose	0.5
R09	Symptoms/complaints sinuses	1
R73	Furuncle/abscess nose	2
R74	Acute upper respiratory tract infection	2
R74.01	Common cold	1
R96	Asthma	2
R90	Hypertrophy/chronic infection tonsils/adenoid	2
R72	Streptococcal pharyngitis/red spark	1
R72.01	Streptococcal pharyngitis	1
R72.02	Red spark	1
R74.02	Acute pharyngitis	1
R75	Acute/chronic rhinosinusitis	2
R75.01	Acute rhinosinusitis	2
R75.02	Chronic rhinosinusitis	2
R76	Acute tonsillitis/peritonsillar abscess	2
R76.01	Acute tonsillitis	2
R76.02	Peritonsillar abscess	1
R77	Acute laryngitis/tracheitis	2
R77.01	Subglottic laryngitis/pseudo croup	1
R77.02	Acute epiglottitis	1
R78	Acute bronchitis/bronchiolitis	2
R81	Pneumonia	3
R91	Chronic bronchitis/bronchiectasis	1
R91.01	Chronic bronchitis	1
R91.02	Bronchiectasis	4
Gastro-intestinal complaints^c^		
D11	Diarrhea	2
D70	Infectious diarrhea, dysentery	1
D70.01	Salmonella	1
D70.02	Shigella-/Yersinia-/Campylobacter intestinal infection	2
D70.03	Giardia	2
D73	Presumed gastro-intestinal infection	2
D86	Other peptic ulcer	1
D93	Inflammatory bowel syndrome	1
D94	Ulcerative colitis/chronic enteritis	1
D94.01	Ulcerative colitis	1
Other infections^d^		
L70.01	Osteomyelitis	1
L70.02	Septic arthritis	1
N71	Meningitis/encephalitis	2
N71.01	Bacterial meningitis	2
N71.02	Viral meningitis	2
N71.03	Encephalitis	2
N71.04	Myelitis	2
Auto-immune symptoms^d^		
B04	Symptoms/complaints blood/blood forming organs	1
B81	Pernicious/folic acid anemia	1
B82	Other/non specified anemia	1
B83	Purpura/coagulation disorder/aberrant thrombocytes	1
B83.02	Idiopathic thrombocytopenic purpura (ITP)	2
L88	Rheumatoid arthritis/related diseases	1
L88.01	Rheumatoid arthritis	2
R83.02	Sarcoidosis	1
S23.01	Alopecia areata	1
S99.04	Vitiligo	1
T86	Hypothyroidism	1
T99.02	Thyroiditis	1
T99.12	Adrenal insufficiency	1
D94.02	Crohn’s disease	2
D99.06	Coeliac disease	1
N99	Myasthenia gravis	1

Malignancies, lymphoproliferative- and other symptoms^d^		
ICPC code	Description	Score
D74	Gastric cancer	1
B72	Hodgkin’s disease	1
B72.01	Hodgkin’s disease	1
B72.02	Non-Hodgkin lymphoma	2
T08	Weight loss	1
B87	Splenomegaly	2
B02	Lymphadenopathy	1
D96	Hepatomegaly	1
T10	Failure to thrive	2
A04	Fatigue/weakness	1
B84	Aberrant leukocytes	0.5
N94	Other peripheral neuritis/neuropathy	1

Laboratory values^e^		
Laboratory measure	Aberrant value (g/L)	Score if aberrant value present
IgG-total	< 7	8
IgG1	< 4.9	8
IgG2	< 1.5	8
IgG3	< 0.2	8
IgG4	< 0.08	8
IgM-total	< 0.4	8
IgA-total	< 0.7	4
Calculated globulin (total protein − albumin)	< 18	6

Visits to general practitioner^f^		
Description	Cut-off value	Score if visits ≥ cut-off value
Visits to the general practitioner clinic in the year before the censoring date	≥ 6 visits	3

Ambiguous codes^g^		
ICPC code	Description	
B72	Hodgkin’s lymphoma	
B72.01	Hodgkin’s lymphoma	
B72.02	Non-Hodgkin lymphoma	
A87.02	Post-transplantation	
D74	Gastric cancer	
D74	Colon or rectal cancer	

Exclusion criteria		
ICPC code/other	Description	
B73	Leukemia	
B74.01	Multiple myeloma	
B90	HIV-infection	
B90.01	HIV seropositive without symptoms	
B90.02	AIDS/AIDS-related complex	
P15.01	Alcoholism	
P15.02	Delirium tremens	
P15.03	Wernicke–Korsakoff	
P19.03	Addiction to hard drugs	
T06	Anorexia nervosa/bulimia	
T06.01	Anorexia nervosa	
T06.02	Bulimia	
T99.01	Immunodeficiency	
T99.10	Cystic fibrosis	
Age	< 12 years or > 70 years	

*AIDS* acquired immune deficiency syndrome*, ATC* anatomic therapeutic chemical, *EHR* electronic health record, *HIV* human immunodeficiency virus, *ICPC* International Classification of Primary Care, *Ig* immunoglobulin

^a^Total score in the 4 years before the censoring date is calculated. If a patient is enrolled in the GP clinic less than 4 years, this is corrected for with the formula: (total number of prescriptions * 4)/(number of days enrolled/365). Prescriptions before the age of 6 years are not taken into account

^b^Score is attributed if the ICPC code is registered in the 10 years before the censoring date

^c^Score is attributed if the ICPC code is registered in the 10 years before the censoring date. Auto-immune symptoms are registered below

^d^Score is attributed if the ICPC code is registered in the EHR at any time-point before the censoring date

^e^Score is attributed if a reduced lab value is registered in the EHR at any time-point before the censoring date

^f^Both physical and electronic visits are taken into account

^g^For these codes, EHRs were screened up to the moment of the ambiguous diagnosis

## Discussion

In the current study we have developed a screening algorithm to identify patients at risk of PAD in a primary care setting, based on EHR data. The components of the algorithm encompass a broad range of presenting signs and symptoms of PAD, including antibiotic prescriptions, diagnostic ICPC codes, laboratory values and the number of visits to the GP. The presented algorithm can be used as an aid to notify GPs when further laboratory evaluation of immunoglobulins should be considered, and thus has the potential to considerably reduce the diagnostic delay of PAD.

Our results show a clear distinction in the number of antibiotic prescriptions when comparing PAD patients with relevant control groups. This effect was especially pronounced in the 4 years before PAD diagnosis. Furthermore, our results show a distinction regarding the number of GP consultations per year. This is in line with the main results from a previous study that compared primary care date of CVID patients with controls [46]. This study also found an increased odds ratio regarding the number of requests for lung function tests and blood tests for infection, a finding which could not be reproduced in the current study. However, the authors reported that a low effectiveness was expected of these measures as a screening tool, due to either a low procedure occurrence among cases or a relatively high occurrence of these procedures amongst controls. Lastly, our results show that serum immunoglobulin levels were very rarely requested by GPs. This could indicate that GPs do not often consider the PAD diagnosis, or that they are not yet aware of the added value of assessing immunoglobulin levels. The implementation of the algorithm presented in this study could aid GPs in when to consider requesting serum immunoglobulin levels, or calculated globulin as an alternative.

As reducing the diagnostic delay of PAD is of great clinical interest, several other studies have investigated screening possibilities for PAD and other PIDs. Several sets of early warning signs (EWS) have been developed by, for example, the Jeffrey Modell Foundation (JMF) and the European Society of Immunodeficiencies. However, these have been shown to have a poor performance, especially in adults [23–26, 28]. This could partially be because these warning signs focus almost exclusively on infectious complications, rather than, for instance, auto-immune symptoms. In addition, these manual screening systems are suboptimal for the detection of rare and heterogeneous diseases, because they depend on the awareness of an individual treating physician. For this reason, efforts have been made to develop automated algorithms for the early detection of PID such as the “SPIRIT software” of the JMF, the “PI Prob”, and a third which is currently in development [52–54]. Of these, only the “PI Prob” has been internally validated. These efforts are of great importance, however these algorithms have been developed mainly for secondary care, and mostly based on pediatric data. These may be less suitable for the recognition of PAD, as most patients present in adulthood [9–12]. Therefore, these pediatric and secondary care algorithms could be of complementary value to the primary care algorithm presented in this paper.

In this study, we focused specifically on the early detection of PAD in primary care. As mentioned, the advantage of primary care EHRs is that they include a comprehensive overview of medical complaints. In addition, patients often primarily present at the GP, thus allowing for an early recognition of high-risk patients. Focusing on PAD, rather than the full spectrum of PIDs, allows use of this algorithm in combination with serologic testing for immunoglobulins. Serologic testing for the full spectrum of PIDs would entail more expensive tests that are difficult to request and interpret by GPs, such as vaccination responses. By using the current algorithm to identify patients at risk for PAD for whom immunoglobulin analysis is indicated, it is both feasible and affordable to implement in primary care.

Because PAD are rare, our study was limited by a small sample size of PAD patients. In an effort to increase the sample size, we also analyzed primary care patients with a diagnostic code for immunodeficiency (T99.01). This group is expected to approximate PAD patients, as causes of secondary immunodeficiencies were excluded and because PAD represents the majority of the PIDs. Therefore, this group was considered to be of interest, in addition to the group of confirmed PAD patients. A second limitation was that we only had access to metadata (e.g. means/medians) from the JHN, due to privacy regulations. We could therefore only evaluate the diagnostic accuracy of individual components, rather than combinations of different components.

As we aimed to design an algorithm that can easily be automated, we focused on structured EHR data. The components of the algorithm are therefore inherently dependent on the available structured EHR data, such as diagnostic codes and medication prescriptions. Certain factors of possible interest, such as the number of hospitalizations, were unfortunately not structurally available in primary care. Furthermore, we did not have access to the data regarding the number of visits to the GP of the PAD patients from the academic hospital. These data were available for the primary care immunodeficiency patients. Although this is a limitation, it is important to note that the number of visits to the GP is only 1 of the 106 components of the algorithm.

Our algorithm is designed to detect adolescent and adult patients aged 12–70 years at risk of undiagnosed PAD in a primary care setting. We focused on patients aged ≥ 12 years, as other cut-off points are to be expected for younger patients (e.g. due to frequent infections and antibiotic prescriptions), and because most PADs present in adulthood [9–12]. Consequently, our algorithm may be less suitable to detect XLA patients, as these usually present with symptoms during the first few years of life. However, our main aim is to reduce the diagnostic delay of PAD, which is considerably lower for XLA patients (e.g. reported median of 1 year for XLA vs. 7.5 years for PAD in general [12]), and almost all cases of XLA are diagnosed before 5 years of age [55]. In addition, it has recently been suggested to add XLA to newborn screening, which is likely a more effective way to reduce diagnostic delay for this particular PAD [56]. Lastly, the diagnostic delay in our cohort was relatively high (median 9.5 years), which may be a consequence of our focus on a population aged 12–70 years, although the delay is within the ranges described in previous cohorts of PAD patients [12, 13].

A major strength of the current study is that our algorithm is based completely on structured EHR data, making it suitable to implement in an automated manner. As ICPC and ATC codes are used internationally, this algorithm could potentially also be applied in other countries. In a consecutive study, we will prospectively validate the algorithm by applying it to 60,000 primary care EHRs in the Netherlands. The highest scoring patients will undergo laboratory assessment of serum immunoglobulin levels, and referral to an immunologist if necessary. This prospective study will allow analysis of patient-level data and determine the optimal cut-off value for high-risk patients.

## Conclusions

In conclusion, in this study we developed a screening algorithm based on presenting signs and symptoms of PAD that is suitable to implement in primary care. It has the potential to considerably reduce the diagnostic delay of PAD, and will be evaluated in a prospective study.

## Supplementary Information

**Additional file 1: Table S1.** Youden’s index per ICPC code.

**Additional file 2: Table S2.** Mean number of antibiotic prescriptions per ATCcode in the 4 years before the extraction date.

**Additional file 3: Figure S1.** Data from the primary care electronic health record (EHR) on the number of diagnostic requests for leukocytes, C-reactive protein (CRP) and lung function tests. The censoring date is the date before which the EHR was screened. For most patients this is the date of data-extraction, November 2021. For immunodeficiency patients pre-diagnosis, the censoring date is the diagnosis date. For details on the censoring date, see “Methods” section.

## Data Availability

Data are handled according to the FAIR principles. For access to the study data, an application can submitted to the corresponding author, which will be reviewed by the study team.

## Abbreviations

ATC: Anatomical Therapeutic Chemical
COPD: Chronic obstructive pulmonary disease
CRP: C-reactive protein
CVID: Common variable immunodeficiency
EHR: Electronic health record
GP: General practitioner
IBD: Inflammatory bowel disease
Ig: Immunoglobulin
ICPC: International Classification of Primary Care
JHN: Julius General Practitioner’s Network
JMF: Jeffrey Modell Foundation
PAD: Primary antibody deficiencies
PID: Primary immunodeficiency
RTI: Respiratory tract infection
SpAD: Specific antibody deficiency
XLA: X-linked agammaglobulinemia
