# Supplementary material for: Development of a primary care screening algorithm for the early detection of patients at risk of primary antibody deficiency
Source: Allergy Asthma Clin Immunol. 2023 May 27;19:44. doi: 10.1186/s13223-023-00790-7 (PMC10224324; doi:10.1186/s13223-023-00790-7)
Supplement: Supplementary file 1 — Additional file 1: Table S1. Youden’s index per ICPC code. [file 13223_2023_790_MOESM1_ESM.docx]

**Table S1** Youden’s index per ICPC code

*Youden’s index was calculated based on the presence of each code in the patient groups with primary antibody deficiency versus the control group. A higher index indicates a higher discriminative value of the ICPC code. ICPC: International Classification of Primary Care.*

| **ICPC code** | **Description** | **Youden’s index** |
| --- | --- | --- |
| A04 | Tiredness/weakness | 10 |
| A91.07 | Subclinical hypothyroidism | 1.5 |
| B02 | Enlarged lymph nodes | 16 |
| B04 | Symptoms / complaints blood / blood forming organs | 2 |
| B72 | Hodgkin’s disease | 0 |
| B72.01 | Hodgkin’s disease | 0 |
| B72.02 | Non-hodgkin lymphoma | 3.5 |
| B81 | Pernicious / folic acid anemia | 0 |
| B82 | Other / non specified anemia | 4 |
| B83 | Purpura / coagulation disorder / aberrant thrombocytes | 0 |
| B83.02 | Idiopathic thrombocytopenic purpura (ITP) | 3.5 |
| B84 | Aberrant leukocyte count | 0 |
| B87 | Splenomegaly | 15 |
| D11 | Diarrhea | 17.5 |
| D70 | Infectious diarrhea, dysentery | -1 |
| D70.01 | Salmonella | 0 |
| D70.02 | *Shigella-/Yersinia-/Campylobacter-*intestinal infection | 1.5 |
| D70.03 | Giardia | 1.5 |
| D73 | Presumed gastro-intestinal infection | 2 |
| D74 | Gastric cancer | 0 |
| D77.01 | Malignancy esophagus | 0 |
| D86 | Other peptic ulcer | 2 |
| D86.01 | Ventricular ulcer | 0 |
| D93 | Inflammatory Bowel Syndrome | 4 |
| D94 | Ulcerative colitis / chronic enteritis | 0 |
| D94.01 | Ulcerative colitis | 0 |
| D94.02 | Crohn’s disease | 7 |
| D99.06 | Celiac disease | 0 |
| H01 | Pain in ear | 5.5 |
| H04 | Discharge from ear | 2.5 |
| H70 | External otitis | 0.5 |
| H71 | Acute otitis media / myringitis | 16 |
| H72 | Otitis media with effusion | 7 |
| H74 | Chronic otitis media / other ear infection | 8.5 |
| H74.01 | Chronic otitis media | 1.5 |
| H74.02 | Mastoiditis | 0 |
| L70 | Infectious disease of the musculoskeletal system | 0 |
| L70.01 | Osteomyelitis | 0 |
| L70.02 | Septic arthritis | 0 |
| L88 | Rheumatoid arthritis / related diagnoses | 4 |
| L88.01 | Rheumatoid arthritis | 0 |
| N71 | Meningitis / encephalitis | 8.5 |
| N71.01 | Bacterial meningitis | 0 |
| N71.02 | Viral meningitis | 0 |
| N71.03 | Encephalitis | 0 |
| N71.04 | Myelitis | 0 |
| N94 | Other peripheral neuritis / neuropathy | 1 |
| N99 | Myasthenia gravis | 0 |
| R05 | Coughing | 39.5 |
| R07 | Sneezing / congested nose / running nose | 0 |
| R09 | Symptoms / complaints sinuses | 1.5 |
| R72 | Streptococcal pharyngitis / red spark | -1 |
| R72.01 | Streptococcal pharyngitis | 0 |
| R72.02 | Red spark | 0 |
| R73 | Furuncle / abscess nose | 2 |
| R74 | Acute upper respiratory tract infection | 44.5 |
| R74.01 | Common cold | 5.5 |
| R74.02 | Acute pharyngitis | -4 |
| R75 | Acute / chronic rhinosinusitis | 12.5 |
| R75.01 | Acute rhinosinusitis | 11 |
| R75.02 | Chronic rhinosinusitis | 20 |
| R76 | Acute tonsillitis / Peritonsillar abscess | 2.5 |
| R76.01 | Acute tonsillitis | 4 |
| R76.02 | Peritonsillar abscess | 0 |
| R77 | Acute laryngitis / tracheitis | 3 |
| R77.01 | Subglottic laryngitis / pseudo croup | 0 |
| R77.02 | Acute epiglottitis | 0 |
| R78 | Acute bronchitis/bronchiolitis | 8 |
| R81 | Pneumonia | 20.5 |
| R83.02 | Sarcoidosis | 3.5 |
| R90 | Hypertrophy / chronic infection tonsils / adenoid | 10 |
| R91 | Chronic bronchitis / bronchiectasis | 0 |
| R91.01 | Chronic bronchitis | 0 |
| R91.02 | Bronchiectasis | 10 |
| R95 | COPD (chronic obstructive pulmonary disease) | -1 |
| R96 | Asthma | 9 |
| S23.01 | Alopecia areata | 2.5 |
| S99.04 | Vitiligo | 3.5 |
| T08 | Weight loss | 5.5 |
| T10 | Failure to thrive | 4 |
| T86 | Hypothyroidism | 2.5 |
| T90.01 | Diabetes mellitus type 1 | 2 |
| T99.02 | Thyroiditis | 0 |
| T99.12 | Adrenal gland insufficiency | 0 |
