# Supplementary material for: Development of a primary care screening algorithm for the early detection of patients at risk of primary antibody deficiency
Source: Allergy Asthma Clin Immunol. 2023 May 27;19:44. doi: 10.1186/s13223-023-00790-7 (PMC10224324; doi:10.1186/s13223-023-00790-7)
Supplement: Supplementary file 2 — Additional file 2: Table S2. Mean number of antibiotic prescriptions per ATCcode in the 4 years before the extraction date. [file 13223_2023_790_MOESM2_ESM.docx]

**Table S2** Mean number of antibiotic prescriptions per ATC (Anatomical Therapeutic Chemical) code in the 4 years before the extraction date

| **ATC-code** | **Description** | **Control group (mean)** | **Immunodeficiency**  **pre-diagnosis (mean)** | **Difference in means** |
| --- | --- | --- | --- | --- |
| J01AA02 | Doxycycline | 0.02 | 0.05 | 0.03 |
| J01CA04 | Amoxicillin | 0.03 | 0.15 | 0.12 |
| J01CE02 | Phenoxymethylpenicillin | 0 | NA | NA |
| J01CE05 | Pheneticillin | 0 | 0.08 | 0.08 |
| J01CF05 | Flucloxacillin | 0.01 | 0.03 | 0.02 |
| J01CR02 | Amoxicillin / Clavulanic acid | 0.01 | 0.12 | 0.11 |
| J01DB01 | Cefalexin | 0 | NA | NA |
| J01DC02 | Cefuroximaxetil | 0 | NA | NA |
| J01DC04 | Cefaclor | 0 | NA | NA |
| J01DD14 | Ceftibuten | 0 | NA | NA |
| J01EE01 | Cotrimoxazole | 0 | 0.19 | 0.19 |
| J01FA01 | Erythromycin | 0 | NA | NA |
| J01FA09 | Clarithromycin | 0 | 0.02 | 0.02 |
| J01FA10 | Azithromycin | 0.01 | 0.21 | 0.2 |
| J01MA02 | Ciprofloxacin | 0.01 | 0.03 | 0.02 |
| J01MA12 | Levofloxacin | 0 | NA | NA |
| J01MA14 | Moxifloxacin | 0 | 0.03 | 0.03 |
| J01XD01 | Metronidazole | NA | NA | NA |
| S02AA16 | Ofloxacin ear suspension | 0 | NA | NA |
| S02CA03 | Hydrocortisone / colistin / bacitracin ear suspension | 0.01 | 0 | -0.01 |
