# Supplementary material for: Development of a primary care screening algorithm for the early detection of patients at risk of primary antibody deficiency
Source: Allergy Asthma Clin Immunol. 2023 May 27;19:44. doi: 10.1186/s13223-023-00790-7 (PMC10224324; doi:10.1186/s13223-023-00790-7)
Supplement: Supplementary file 3 — Additional file 3: Figure S1. Data from the primary care electronic health record (EHR) on the number of diagnostic requests for leukocytes, C-reactive protein (CRP) and lung function tests. The censoring date is the date before which the EHR was screened. For most patients this is the date of data-extraction, November 2021. For immunodeficiency patients pre-diagnosis, the censoring date is the diagnosis date. For details on the censoring date, see “Methods” section. [file 13223_2023_790_MOESM3_ESM.docx]

**Figure S1**

*Data from the primary care electronic health record (EHR) on the number of diagnostic requests for diagnostic requests for leukocytes, C-reactive protein (CRP) and lung function tests. The censoring date is the date before which the electronic health care record (EHR) was screened. For most patients this is the date of data-extraction, November 2021. For immunodeficiency patients pre-diagnosis, the censoring date is the diagnosis date. For details on the censoring date, see the Methods section.*

A


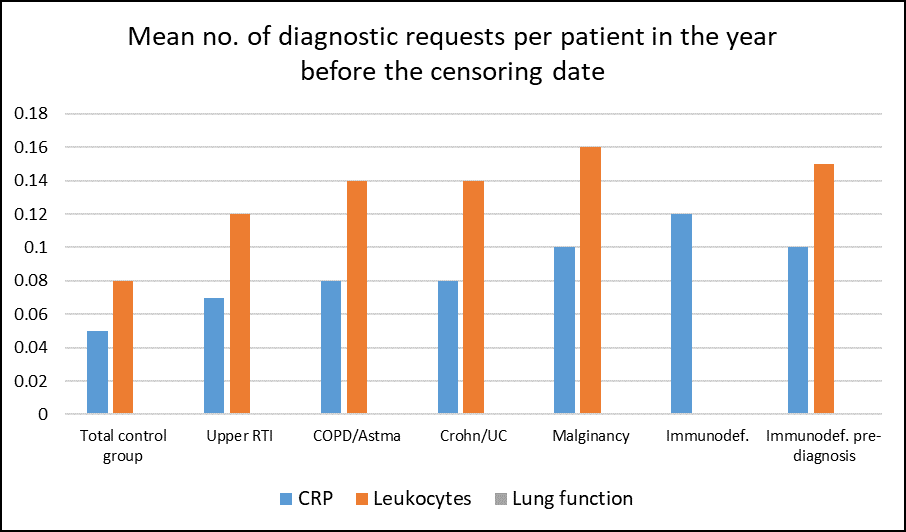


**Fig. S1A** Mean number of diagnostic requests per year for different patient populations. The mean number of requested lung function tests per patient in the year before the censoring date was too low to be visible in this figure. There is no clear distinction between the non-specified immunodeficiency patients pre-diagnosis and the other control groups, indicating that these parameters are not of additional value to the algorithm.
COPD chronic obstructive pulmonary disease, CRP C-reactive protein, immunodef. immunodeficiency patients from primary care, no. number, RTI respiratory tract infection.

B


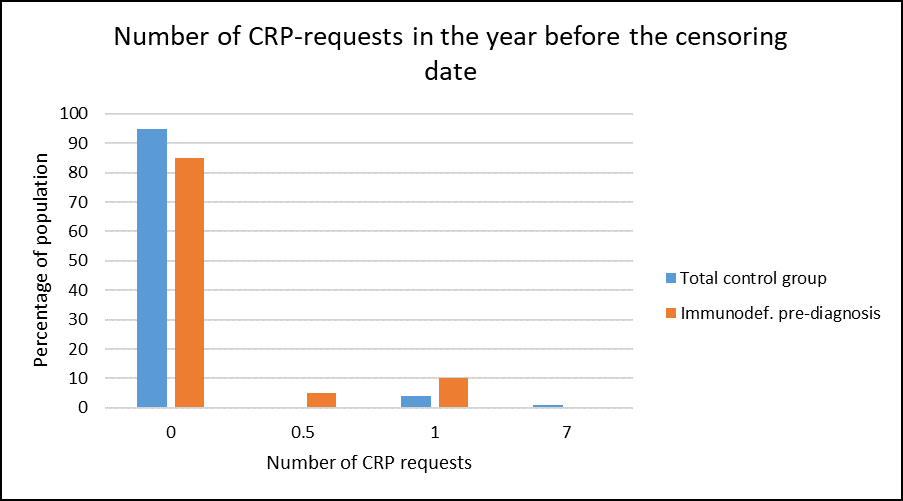


**Fig. S1B** The proportion of the population is shown with respect to the number of requests for CRP in the year before the censoring date. No clear distinction is visible between the general population and the immunodeficiency patients pre-diagnosis, indicating that adding this parameter to the algorithm is of no additional value.
CRP C-reactive protein, immunodef. immunodeficiency patient from primary care.

**Fig. S1C** The proportion of the population is shown with respect to the number of requests for leukocytes in the year before the censoring date. No clear distinction is visible between the general population and the immunodeficiency patients pre-diagnosis, indicating that adding this parameter to the algorithm is of no additional value. Immunodef immunodeficiency patients from primary care.


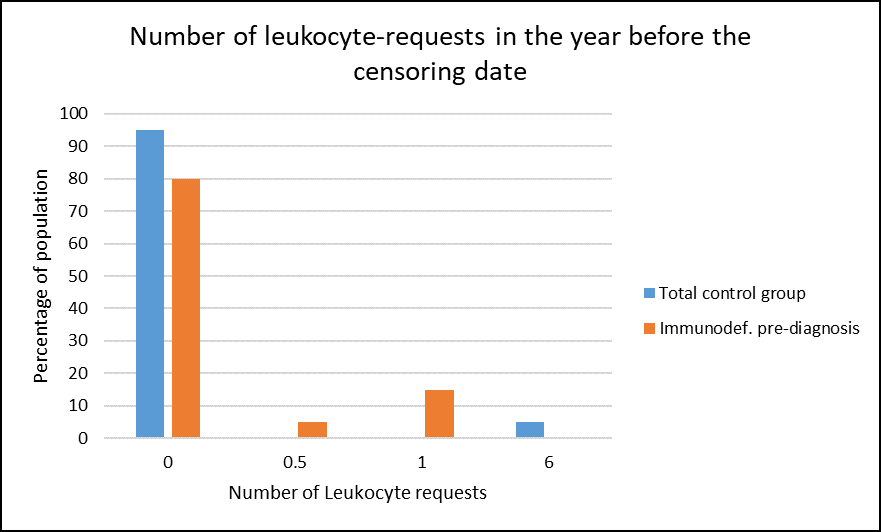


C

D


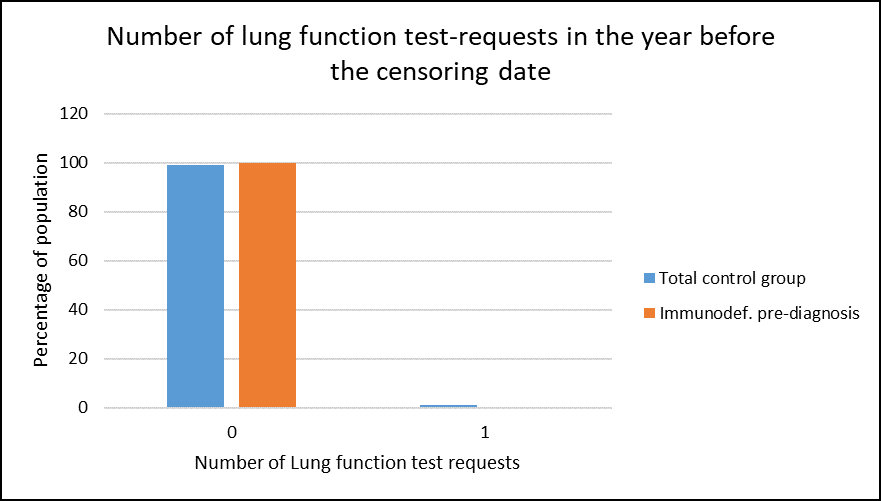


**Fig. S1D** The proportion of the population is shown with respect to the number of requests for lung function tests in the year before the censoring date. No clear distinction is visible between the general population and the immunodeficiency patients pre-diagnosis, indicating that adding this parameter to the algorithm is of no additional value. Immunodef: immunodeficiency patients from primary care.
